# Hepatitis C cell culture system – a valuable tool for understanding viral resistance mechanisms

**DOI:** 10.1186/1471-2334-14-S4-P22

**Published:** 2014-05-29

**Authors:** Costin-Ioan Popescu, Ovidiu Vlaicu, Leontina Bănică, Simona Paraschiv, Dan Oțelea

**Affiliations:** 1 Institute of Biochemistry of the Romanian Academy, Bucharest, Romania; 2 National Institute for Infectious Diseases “Prof. Dr. Matei Balş”, Bucharest, Romania

## 

Human Immunodeficiency Virus (HIV) and Hepatitis C Virus (HCV) co-infect five million people worldwide. HIV-HCV co-infection represents the major cause of liver related morbidity among the HIV infected patients. The current HCV standard of care consisting of pegylated α-interferon and ribavirin has variable efficacy and considerable side-effects. A major step towards a more efficient and better tolerated therapy has been done by the recent approval of the first direct acting antivirals (DAAs) to enter the clinic. Moreover, there are numerous DAAs which are advanced in clinical trials targeting different steps in the viral life cycle. Due to the variety and efficacy of HCV drugs in the clinic or in the pipeline, there is hope for an interferon free treatment. However, despite the increased efficacy of DAAs with or without interferon and ribavirin, viral resistance still represents an issue. Thus, phenotyping resistance “in vitro” should help the clinician to better personalize the HCV treatment. HCV drug resistance phenotyping has become possible due to the HCV cell culture system.

HCV cell culture (HCVcc) system relies on the JFH-1 strain of genotype 2a which is able to replicate in cell culture without adaptive mutations. HCVcc system allows the analysis of every step in the viral life cycle: replication, secretion and infectivity. Clinically relevant chimeric viruses are available covering different HCV genotypes. This allows the “in vitro” evaluation of the viral fitness starting from patient derived viral sequences. We are presenting the complementarity between drug resistance genotyping and HCVcc system in understanding the mechanisms of HCV drug resistance. The use of a drug resistance phenotyping assay for clinical purpose will be discussed.

